# Susceptibility-Weighted Breast MRI Differentiates Abscesses from Necrotic Tumors: A Prospective Evaluation

**DOI:** 10.3390/diagnostics15172260

**Published:** 2025-09-07

**Authors:** Fadime Güven, Muhammed Halid Yener

**Affiliations:** Department of Radiology, Faculty of Medicine, Atatürk University, 25240 Erzurum, Türkiye; mhalidyener@gmail.com

**Keywords:** breast, abscess, necrotic tumor, magnetic resonance imaging, susceptibility-weighted imaging

## Abstract

**Background/Objectives**: Breast abscesses and necrotic masses often show similar peripheral enhancement and a fluid-containing appearance on breast MRI, leading to diagnostic confusion. Accurate differentiation is critical because biopsies that fail to sample the lesion wall may yield false-negative results, may be misinterpreted as an infectious process, and delay diagnosis. Incorporating SWI into the protocol can provide additional clues to malignancy and, when warranted, prompt a second wall-targeted biopsy, thus reducing the risk of delayed cancer diagnosis. **Methods**: This single-center prospective diagnostic accuracy study included 42 female patients diagnosed between 2022 and 2025 with either necrotic breast tumors or abscesses, confirmed by histopathology. SWI-based Intralesional Susceptibility Score (ILSS), rim morphology, and mean ADC values were evaluated. Statistical analyses included the Mann–Whitney U test, chi-square test, ROC analysis, DeLong test for comparison of AUCs, and Cohen’s kappa for interobserver agreement. **Results**: SWI-based ILSS values were significantly higher in necrotic tumors compared to abscesses (mean ILSS: 2.28 vs. 0.85; 95% CI: 1.0–2.0; *p* < 0.001). Smooth hypointense rims were predominantly observed in abscesses (Sensitivity: 63.1%, 95% CI: 0.38–0.83; Specificity: 88.9%, 95% CI: 0.65–0.98; *p* = 0.001). Incomplete rim morphology was more frequent in tumors (Sensitivity: 78.9%, 95% CI: 0.54–0.93; Specificity: 77.8%, 95% CI: 0.52–0.93; *p* < 0.001). The double rim sign was highly specific for abscesses (Specificity: 95.2%, 95% CI: 0.76–0.99 *p* = 0.002). **Conclusions**: SWI provides valuable morphological information in differentiating abscesses from necrotic tumors on breast MRI. When used in combination with ADC values, it can enhance diagnostic accuracy.

## 1. Introduction

Breast MRI is increasingly used as a problem-solving modality. It is recommended for screening high-risk women—those with genetic mutations, a significant family history, prior chest irradiation at a young age, or a personal history of breast cancer—who are at elevated risk [1]. MRI also aids in assessing multicentricity in newly diagnosed breast cancer, guiding surgical planning, and evaluating residual tumor and treatment response.

However, certain cystic–necrotic malignant lesions and abscesses arising in an inflammatory background—particularly those associated with idiopathic granulomatous mastitis—may show substantial overlap in imaging features, including irregular morphology, prominent rim enhancement, and suspicious dynamic contrast-enhancement kinetics (plateau or washout), thereby complicating accurate differentiation [2,3,4,5,6,7]. At the same time, in malignant lesions presenting with cystic-necrotic characteristics, failure to sample the lesion wall increases the risk of false-negative results and may necessitate a repeat biopsy, with magnetic resonance imaging findings playing a critical role in guiding this decision [8,9]. Given these diagnostic challenges, advanced imaging techniques such as Susceptibility-Weighted Imaging (SWI) may offer additional diagnostic information.

T2-weighted imaging based on spin-echo sequences is designed to minimize magnetic susceptibility artifacts. In contrast, three-dimensional Susceptibility-Weighted Imaging (SWI), which is based on T2*-weighted gradient-echo sequences, is intentionally sensitive to paramagnetic and diamagnetic substances that distort the local magnetic field.

SWI effectively utilizes phase information, which is often overlooked in conventional MRI. In phase imaging, paramagnetic substances cause positive phase shifts, whereas diamagnetic substances result in negative phase shifts. The phase images obtained in this technique are processed through filtering to generate a phase mask. This mask is multiplied by the magnitude image to produce the final SWI image. Through this approach, microhemorrhages, fine venous structures, and areas of calcification can be visualized with high tissue contrast.

In neuroimaging, SWI is widely used in various clinical settings, such as traumatic brain injury, neurodegenerative diseases, and differentiation of tumors and metastases. Additionally, previous studies have shown that SWI can differentiate brain abscesses from necrotic masses [10,11,12].

SWI offers advantages in both enhancing the visibility of the peripheral hypointense rim and in characterizing its morphology. Furthermore, components such as hemosiderin (paramagnetic) and calcification (diamagnetic), frequently observed in necrotic tumors, can be visualized with high specificity due to SWI’s susceptibility-based contrast mechanism.

The necrosis-to-wall ADC ratio on breast MRI has been proposed as a method for differentiating necrotic malignant masses from abscesses [13]. However, evidence indicates that DWI, when used in isolation, may be insufficient in differentiating these entities in some cases [6,14]. Nevertheless, studies exploring the role of susceptibility-weighted imaging (SWI) in breast MRI remain limited, with existing literature predominantly focusing on the detection of microcalcifications [15,16,17]. To the best of our knowledge, no prior publication has evaluated the diagnostic contribution of SWI in distinguishing necrotic breast lesions from abscesses; therefore, this study represents a novel contribution to the field.

In recent years, studies on the diagnostic performance of non-contrast breast MRI have accelerated [18,19]. In this context, our study anticipates that SWI may provide additional value in reducing the limitations of evaluations performed without the use of a contrast agent. At the same time, the absence of a requirement for contrast administration, coupled with a short additional acquisition time (approximately 2–2.5 min), supports the feasibility of incorporating SWI into standard breast MRI protocols.

The aim of this study is to evaluate the diagnostic performance of SWI sequences in differentiating abscesses from necrotic tumors on breast MRI and to assess whether combining SWI findings with ADC values improves diagnostic accuracy.

## 2. Materials and Methods

### 2.1. Patients

This prospective diagnostic accuracy study was conducted in accordance with the Declaration of Helsinki, national regulations on human rights, and Good Clinical Practice (GCP) guidelines. Ethical approval was obtained from the local institutional review board (Atatürk University Faculty of Medicine, Non-Interventional Clinical Research Ethics Committee; Approval No: B.30.2.ATA.0.01.00/66, 29 December 2022). Written informed consent was obtained from all participants prior to MRI and biopsy procedures. Between December 2022 and July 2025, female patients who were referred for breast MRI due to clinical or imaging (mammography or sonography) suspicion of a mass or abscess lesion and who voluntarily consented to participate were prospectively and consecutively enrolled. Exclusion criteria included lesions smaller than 1 cm, lesions showing only non-mass enhancement, examinations with nondiagnostic image quality due to artifacts, and absence of histopathological confirmation.

### 2.2. MR Imaging

All breast MRI examinations were performed using a 3 Tesla MR system (Siemens Skyra, Siemens AG, Erlangen, Germany) with Siemens syngo MR D13 software (Siemens Healthcare, Erlangen, Germany). In addition to the standard breast MRI protocol, a three-dimensional Susceptibility-Weighted Imaging (3D SWI) sequence was acquired in all cases. SWI was acquired in the sagittal plane with separate acquisitions for each breast, using patient-specific auto-minimum TR (TR 27–43 ms; TE 20 ms; flip angle 15°; matrix 200 × 256; voxel 0.9 mm × 0.9 mm × 3.5 mm). Post-processing of SWI images included minimum intensity projection (MinIP), SWI filtering, and phase/magnitude combination steps.

Standard dynamic contrast-enhanced breast imaging was performed using T1-weighted 3D gradient-echo (GRE) sequences. Dynamic imaging consisted of one pre-contrast and five post-contrast phases (TR/TE: 4.17/1.46 ms; flip angle: 10°; matrix size: 384 × 384; voxel size: 1.1 mm × 1.1 mm × 1.0 mm). As the contrast agent, gadobutrol (Gadovist^®^, Bayer Pharma AG, Berlin, Germany) with a concentration of 1.0 mmol/mL was administered intravenously at a dose of 0.1 mmol/kg body weight.

Fat-suppressed T2-weighted images were obtained using a Turbo Inversion Recovery Magnitude (TIRM) sequence (TR/TE: 3440/68 ms; TI: 150 ms; matrix size: 256 × 256; voxel size: 1.1 mm × 1.1 mm × 4.5 mm).

Diffusion-weighted imaging (DWI) sequences were acquired in the axial plane, and apparent diffusion coefficient (ADC) maps were automatically generated by the system (*b*-values: 0, 600, and 1200 s/mm^2^; TR/TE: 6200/63 ms; matrix size: 128 × 128; voxel size: 1.9 mm × 1.9 mm × 4.5 mm).

### 2.3. Image Analysis

The SWI images were independently evaluated in a double-blind manner by two experienced radiologists (G.F. and Y.M), one of whom specialized in breast imaging. All cases were assessed in a randomized sequence with all patient identifiers removed. Interobserver agreement was calculated, and any discrepancies were resolved by joint consensus.

As part of the morphological analysis, the presence of a hypointense rim was first assessed. Peripheral hypointensity relative to the internal signal intensity of the lesion was considered a positive finding. The morphological characteristics of the hypointense rim were classified separately. The rim was evaluated for margin regularity (regular vs. irregular) and for completeness (complete vs. incomplete appearance). In addition, the presence of a hyperintense ring located within the inner border of the hypointense rim—brighter than the overall lesion signal—was also assessed. This finding was defined as the “double rim” sign [11,20].

For each lesion, the SWI-based Intralesional Susceptibility Score (ILSS) was calculated using the quantitative method originally developed by Park et al. for evaluating tumor grade and perfusion parameters in gliomas, and subsequently applied in several other studies [10,12,21]. ILSS categorizes lesions into four groups based on the number of susceptibility foci:

- Group 0: No susceptibility foci
- Group 1: 1–5 punctate and/or fine linear susceptibility foci
- Group 2: 6–10 punctate and/or fine linear susceptibility foci
- Group 3: More than 10 punctate and/or fine linear susceptibility foci

Additionally, each lesion was manually contoured, and the mean apparent diffusion coefficient (ADC) value was calculated.

### 2.4. Statistical Analysis

The index tests employed in this study comprised the SWI-based Intralesional Susceptibility Score (ILSS), evaluated exclusively on SWI sequences; peripheral rim morphology findings derived from SWI; and apparent diffusion coefficient (ADC) measurements obtained from DWI. The reference standard was histopathological evaluation, performed in all cases.

Statistical analyses were performed using Jamovi (version 2.6.44.0), an open-source statistical software based on R [22,23,24]. For ILSS, the effect size (Cohen’s d) estimated from pilot data was 1.30. To guard against potential over-estimation, we conservatively set d = 1.00 for the a priori power calculation (α = 0.05, power = 0.80). This analysis indicated that 34 participants (17 per group) would be sufficient. Our final sample (N = 42) therefore exceeds this requirement. The Shapiro–Wilk test was used to assess the normality of continuous variables. For comparisons between two groups, the Mann–Whitney U test was applied for numerical variables, while the chi-square test was used for categorical variables.

Interobserver agreement was evaluated using squared-weighted Cohen’s kappa for ordinal semi-quantitative variables such as ILSS scoring, and unweighted Cohen’s kappa for nominal categorical variables such as the presence of a hypointense rim; any discrepancies were subsequently resolved by joint consensus between the two radiologists.

To assess diagnostic performance, receiver operating characteristic (ROC) analyses were conducted for ADC, SWI-based ILSS, and a combined model incorporating both parameters. The area under the curve (AUC) values for each variable were compared using DeLong’s test. The diagnostic accuracy of the combined use of ADC and ILSS was assessed using multivariate logistic regression analysis; predicted probabilities from this model were used to generate a ROC curve. Optimal threshold values were determined using the Youden index, and corresponding sensitivity and specificity values were reported with 95% confidence intervals (CIs).

For the following qualitative imaging features—presence of a hypointense rim, rim completeness, rim margin morphology, and the double rim sign—diagnostic metrics including sensitivity, specificity were calculated and reported with 95% CIs. A *p*-value of <0.05 was considered statistically significant for all analyses.

## 3. Results

After applying the inclusion and exclusion criteria, a final cohort of 42 female patients (mean ± SD age: 50.4 ± 12.4 years) was obtained (Table 1). Among them, 21 were diagnosed with abscesses and 21 with necrotic breast tumors.

**Figure 1:**
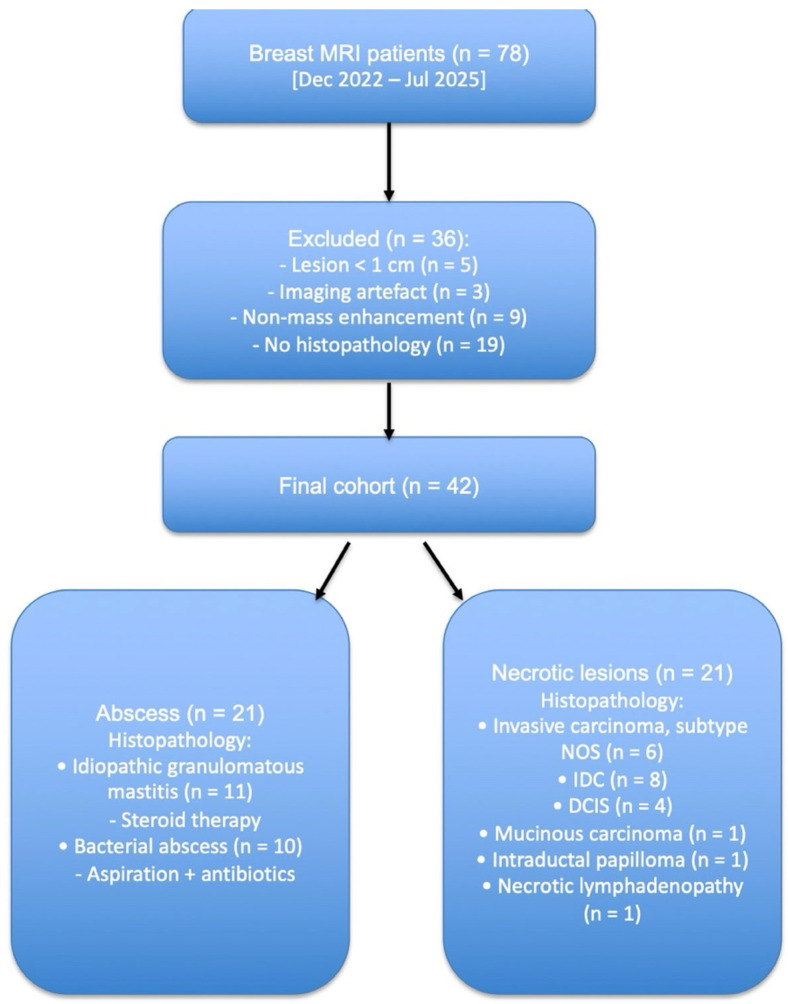
Flow diagram illustrating the patient selection process from the initial 78 cases to the final study cohort.

Histopathological evaluation following tru-cut biopsy revealed 21 lesions with necrotic components. Of these patients, 6 were diagnosed with invasive breast carcinoma of unspecified subtype, 8 with invasive ductal carcinoma (IDC), 4 with ductal carcinoma in situ (DCIS), 1 with mucinous carcinoma, 1 with intraductal papilloma, and 1 with necrotic lymphadenopathy (Figure 1). In all cases, biopsy sampling was specifically targeted to the lesion wall for histopathological analysis.

Among the 21 patients diagnosed with abscesses, 16 were confirmed by tru-cut biopsy and 5 by fine-needle aspiration biopsy (FNAB). Eleven of these patients were diagnosed with idiopathic granulomatous mastitis (IGM) on histopathology. In our clinic, patients with IGM are treated with perilesional steroid injections (Methylprednisolone acetate, Depo-Medrol^®^ 40 mg/mL, Pfizer Inc., New York, NY, USA) [25]. The remaining 10 patients were treated with antibiotics following aspiration, and achieved complete clinical and radiological resolution on follow-up.

Interobserver agreement for ILSS scoring was good, with a squared-weighted Cohen’s κ of 0.60 (95% CI: 0.35–0.85; *p* < 0.001). For morphological categorical findings, the unweighted Cohen’s κ was calculated as 0.77 (95% CI: 0.70–0.84; *p* < 0.001), indicating near-perfect agreement between observers.

The Shapiro–Wilk test was used to assess normality of ADC and ILSS variables. Both variables were found to be non-normally distributed (*p* < 0.001 for ADC, *p* = 0.016 for ILSS). Therefore, comparisons between groups were performed using the Mann–Whitney U test and chi-square test.

A statistically significant difference in mean ADC values was observed between necrotic tumors and abscesses (mean ADC: 0.96 mm^2^/s vs. 0.59 mm^2^/s; 95% CI: 0.10–0.45; *p* = 0.001). Similarly, ILSS scores were significantly higher in necrotic tumors compared to abscesses (mean ILSS: 2.29 vs. 0.86; 95% CI: 1.0–2.0; *p* < 0.001).

**Figure 2:**
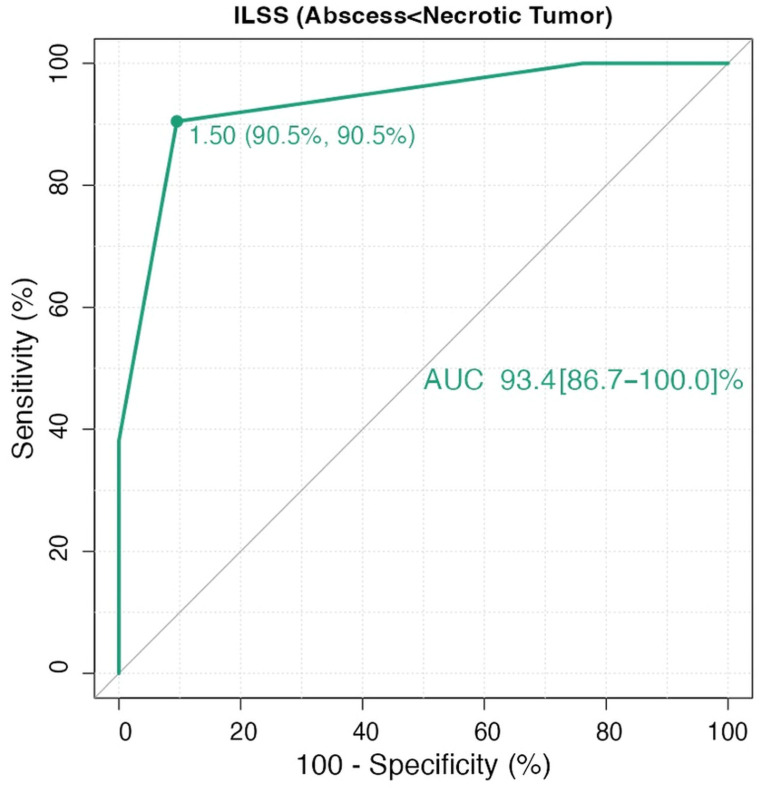
Receiver operating characteristic (ROC) curve demonstrating the diagnostic performance of the SWI-based Intralesional Susceptibility Score (ILSS).

**Figure 3:**
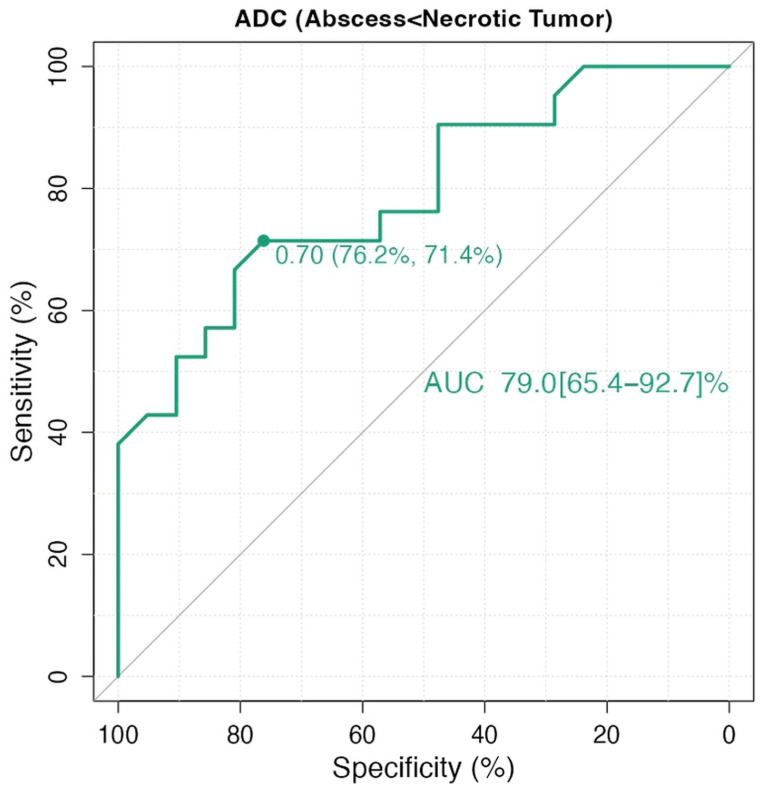
Receiver operating characteristic (ROC) curve for apparent diffusion coefficient (ADC).

ROC analysis for ILSS yielded an AUC of 93.4% (95% CI: 0.86–1.0). Using a threshold of 1.50, the sensitivity and specificity were both 90.5% (Figure 2). For ADC values, ROC analysis showed an AUC of 79.0% (95% CI: 0.65–0.92). An optimal cutoff value of 0.70 mm^2^/s yielded a sensitivity of 76.2% and specificity of 71.4% (Figure 3). In the DeLong test comparison, the AUC difference between the two models was 0.144, which was not statistically significant (95% CI: –0.009 to 0.297; *p* = 0.066).

In the multivariate logistic regression model combining ILSS and ADC parameters, the area under the ROC curve (AUC) was calculated as 97.6% (95% CI: 94.1–100.0). The optimal threshold values for the combined model were determined as 3.0 for ILSS and 0.69 mm^2^/s for ADC. Using these cutoffs, sensitivity was measured at 100.0%, and specificity at 85.7%.

The relationship between morphological parameters assessed on SWI and lesion types (abscess vs. necrotic tumor) was analyzed using the chi-square test. No statistically significant association was found between the presence of a hypointense rim and lesion type (*p* = 1.000). However, rim margin characteristics showed a significant association; smooth margins were more frequently observed in abscesses, while irregular margins were more commonly associated with necrotic tumors (Sensitivity: 63.1%, 95% CI: 0.38–0.83; Specificity: 88.9%, 95% CI: 0.65–0.98; *p* = 0.001). Similarly, a significant association was found between rim completeness and lesion type; incomplete rims were predominantly seen in necrotic tumors, while complete rims were more common in abscesses (Sensitivity: 78.9%, 95% CI: 0.54–0.93; Specificity: 77.8%, 95% CI: 0.52–0.93; *p* < 0.001). Analysis of the double rim sign also demonstrated a significant relationship; this feature was more frequently observed in abscesses (Specificity: 95.2%, 95% CI: 0.76–0.99; Sensitivity: 47.6%, 95% CI: 0.25–0.70; *p* = 0.002) (Table 2 and Table 3).

## 4. Discussion

**Figure 4:**
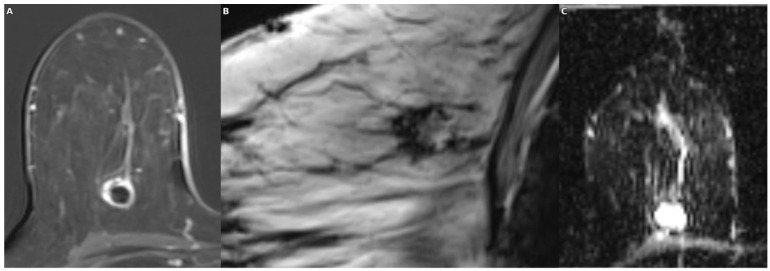
In a patient with ductal carcinoma in situ (DCIS) exhibiting necrotic features, the post-contrast subtraction image (**A**) shows a centrally necrotic mass with peripheral enhancement; the SWI sequence (**B**) demonstrates an irregular hypointense rim encircling the lesion with multiple intralesional susceptibility signal foci; and the ADC map (**C**) reveals relatively high values consistent with facilitated diffusion in the necrotic core.

**Figure 5:**
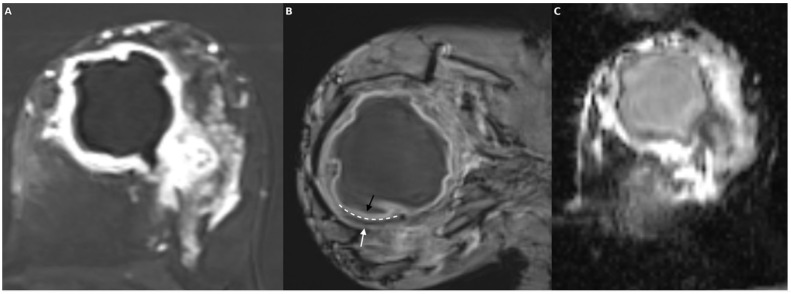
In a patient with an abscess secondary to idiopathic granulomatous mastitis, the post-contrast subtraction image (**A**) shows a rim-enhancing cavity; the SWI sequence (**B**) reveals the characteristic double-rim appearance—an outer hypointense rim indicated by a white arrow and an inner relatively hyperintense rim indicated by a black arrow; and the ADC map (**C**) demonstrates low values consistent with restricted diffusion in purulent content.

Our findings demonstrate that intralesional Susceptibility Score (ILSS) and rim morphology features derived from SWI provide a significant advantage in differentiating abscesses from necrotic tumors, particularly when used in combination with ADC. ILSS alone yielded a very high area under the curve (AUC) of 93.4% with balanced sensitivity and specificity (90.5% each), whereas the moderate discriminative power of ADC (AUC: 79.0%) was markedly enhanced when combined with ILSS, increasing the AUC to 97.6% and sensitivity to 100%. The morphological features derived from SWI further supported these findings: irregular or incomplete rims and the presence of a single-layer rim were more frequently observed in necrotic tumors, whereas smooth, complete, and double-layer rims were more characteristic of abscesses. Notably, the double rim sign showed high specificity for abscesses. Thus, SWI contributed practical value to the diagnostic decision-making process by complementing the limitations of diffusion-weighted imaging through both quantitative (ILSS) and qualitative (rim morphology) indicators in the differentiation of necrosis and abscess (Figure 4 and Figure 5).

On T2-weighted (T2W) imaging, both abscesses and necrotic tumors may present with a peripheral hypointense rim. Previous studies have shown that this finding on T2W images lacks specificity [11]. In contrast, the morphology of the hypointense rim on Susceptibility-Weighted Imaging (SWI) sequences provides more detailed information. One of the most striking findings in our study was the “double rim” sign observed on SWI images. In our statistical analysis, this sign demonstrated a specificity of 95.2% for abscesses, offering considerable discriminatory power in distinguishing abscesses from necrotic tumors. The double rim sign, as described in the neuroradiology literature, refers to two concentric rings surrounding a necrotic cavity: the outer ring appears hypointense, whereas the inner ring demonstrates variable degrees of hyperintensity relative to the cavity contents [11,20]. Our finding parallels the “dual-rim sign” described by C. H. Toh et al. in brain abscesses. Toh and colleagues suggested that the outer hypointense ring corresponds to the fibrous capsule, while the relatively hyperintense inner band reflects granulation tissue and intense inflammatory cell infiltration [11]. We believe a similar histopathological mechanism underlies the imaging features of the breast abscesses in our cohort. In certain abscess cases, the irregular or incomplete hypointense rim and intralesional susceptibility foci may be attributed to idiopathic granulomatous mastitis (IGM), which was present in several patients in our series. Chronic granulomatous inflammation may obscure the typical smooth rim appearance, leading to atypical morphologies on SWI. Tariq et al. provided a detailed description of the progressive loss of lobular architecture across the abscess-ulcerative spectrum in IGM. They noted that during the abscess phase, lobules gradually fuse; in the late ulcerative phase, lobules become indistinct as they coalesce into a single sheet, and in more advanced stages, ducts undergo severe necrosis, ultimately erasing the lobular architecture entirely [26]. This pathological process may account for the imaging findings in our study. The obliteration of lobular boundaries and intense necrotizing granulomatous response may have disrupted the expected smooth hypointense capsule-like rim, reducing sensitivity and resulting in absent or irregular/incomplete rims in some cases. Given the morphological effects of chronic granulomatous inflammation and the well-documented difficulty in distinguishing it from malignancy, our findings suggest that SWI may provide additional diagnostic value in this differentiation [6,14].

In breast cancers, the rapidly proliferating tumor mass leads to the formation of an immature neovascular network to meet increasing demands for oxygen and nutrients. These immature vessels are structurally irregular and highly permeable, making them prone to microhemorrhages. The breakdown of erythrocytes in these areas releases deoxyhemoglobin and hemosiderin, both of which are strongly paramagnetic and appear as punctate or linear hypointense foci on SWI [27]. Additionally, microcalcification foci also contribute to the ILSS. Therefore, the coexistence of rich but disorganized vasculature, microhemorrhage, hemosiderin deposition, and microcalcifications explains the significantly higher ILSS scores observed in necrotic malignant lesions compared to abscesses. Lai et al. [10] combined ILSS scoring with ADC measurements to distinguish necrotic glioblastomas and necrotic metastases from brain abscesses. They demonstrated that this combined model offered superior diagnostic performance compared to either parameter alone [10]. The results from our cohort support this conclusion, confirming that the ILSS + ADC combination yields greater diagnostic accuracy than either parameter in isolation.

Our study has several limitations. First, the overall sample size was relatively small (*n* = 42), and the subgroups (e.g., IGM cases) comprised even fewer cases, which may limit the generalizability of the findings. The Intralesional Susceptibility Score, which has previously been used in neuroimaging, was applied to breast imaging for the first time in this study, which could be considered a methodological weakness. Furthermore, although a power analysis was conducted, the inherent subjectivity of SWI assessment highlights the need for validation of this method in larger, multicenter cohorts. Second, the relatively high prevalence of idiopathic granulomatous mastitis (IGM) cases in our institution may have influenced both the sensitivity of the hypointense rim and the distribution of ILSS scores; therefore, similar results may not be readily replicated in centers with lower IGM prevalence. Third, the extent of histopathological correlation was limited. The inability to directly correlate the double rim sign and ILSS foci with any specific histopathological entities, such as fibrous capsule, granulation tissue, or hemosiderin deposits, represents a significant limitation of the study, and notably, the histopathological correlate of the double rim sign remains controversial. Considering these limitations, our findings require validation through large-scale, multicenter studies.

## 5. Conclusions

This study demonstrates that susceptibility-weighted imaging (SWI) provides significant diagnostic value for the non-invasive differentiation of necrotic malignant breast lesions from abscesses. In cases where biopsy samples are not obtained from the lesion wall in necrotic tumors, there is a risk of false-negative results, potential confusion with infectious pathologies, and consequent diagnostic delays. Therefore, we believe that SWI-based findings may serve as a valuable guide in determining whether a repeat biopsy is necessary in such clinical scenarios. Nevertheless, further validation in larger, multi-institutional cohorts is essential before these findings can be generalized to broader clinical practice.

## Figures and Tables

**Table 1:** Summary of the baseline demographic characteristics of the study sample, including number of patients, mean age ± standard deviation (SD), and gender distribution.

Group	Female Patients (*n*)	Mean Age (Years)	SD
Abscess	21	42.8	8.6
Necrotic lesion	21	58.1	10.8
Total	42	50.4	12.4

**Table 2:** Statistical summary of rim morphology findings.

Feature	*p* Value	Sensitivity % (95% CI)	Specificity % (95% CI)
Hypointense rim	1.00	80.9 (60.0–92.3)	14.3 (5–34)
Rim margin	0.001	63.1 (38–83)	88.9 (65–99)
Rim completeness	<0.001	78.9 (54–93)	77.8 (52–94)
Double rim	0.002	47.6 (25–70)	95.2 (76–99)

**Table 3:** Evaluation of rim features in abscesses and necrotic breast lesions.

Feature	Abscess	Necrotic Tumor
**Hypointense Rim**		
Present	18	18
Absent	3	3
**Rim Margin**		
Irregular	7	16
Smooth	12	2
**Completeness of Rim**		
Incomplete	4	14
Complete	15	4
**Double Rim**		
Absent	11	20
Present	10	1

Bold text indicates the main categories.

## Data Availability

Measurement and evaluation tables, along with the raw statistical datasets, have been deposited in Zenodo under the reserved 10.5281/zenodo.16415544 and are openly available.

## Abbreviations

ILSS	Intralesional Susceptibility Score
